# Colorectal Cancer Early Detection in Stool Samples Tracing CpG Islands Methylation Alterations Affecting Gene Expression

**DOI:** 10.3390/ijms21124494

**Published:** 2020-06-24

**Authors:** Ana Florencia Vega-Benedetti, Eleonora Loi, Loredana Moi, Sandra Orrù, Pina Ziranu, Andrea Pretta, Eleonora Lai, Marco Puzzoni, Letizia Ciccone, Andrea Casadei-Gardini, Francesco Cabras, Federica Fortunato, Angelo Restivo, Luigi Zorcolo, Mario Scartozzi, Patrizia Zavattari

**Affiliations:** 1 Department of Biomedical Sciences, Unit of Biology and Genetics, University of Cagliari, 09042 Cagliari, Italy; anaf.vegab@unica.it (A.F.V.-B.); eleee.loi22@gmail.com (E.L.); lorymoi@gmail.com (L.M.); 2 Department of Pathology, “A. Businco” Oncologic Hospital, ASL Cagliari, 09121 Cagliari, Italy; orrusandra14@gmail.com; 3 Department of Medical Oncology, University Hospital of Cagliari, 09042 Cagliari, Italy; pziranu@libero.it (P.Z.); an.pretta@gmail.com (A.P.); sperimentazioniclinicheunica@gmail.com (E.L.); marcopuzzoni@gmail.com (M.P.); marioscartozzi@gmail.com (M.S.); 4 Bio-Rad Laboratories srl, Southern Europe, 20090 Milan, Italy; letizia_ciccone@bio-rad.com; 5 Division of Oncology, Department of Oncology and Hematology, University Hospital of Modena, 41125 Modena, Italy; casadeigardini@gmail.com; 6 Department of Surgery, Colorectal Surgery Center, University of Cagliari, 09042 Cagliari, Italy; fracab@gmail.com (F.C.); federicafortunato1987@gmail.com (F.F.); arestivo@unica.it (A.R.); lzorcolo@aoucagliari.it (L.Z.)

**Keywords:** Colorectal cancer (CRC), CpG islands, cancer methylation alterations, CRC early diagnosis, biomarkers, *GRIA4*, *VIPR2*, *SPOCK1*, *SLC6A3*, gene downregulation

## Abstract

Colorectal cancer (CRC) is a major cause of cancer mortality. Early diagnosis is relevant for its prevention and treatment. Since DNA methylation alterations are early events in tumourigenesis and can be detected in cell-free DNA, they represent promising biomarkers for early CRC diagnosis through non-invasive methods. In our previous work, we identified 74 early altered CpG islands (CGIs) associated with genes involved in cell cross-talking and cell signalling pathways. The aim of this work was to test whether methylation-based biomarkers could be detected in non-invasive matrices. Our results confirmed methylation alterations of *GRIA4* and *VIPR2* in CRC tissues, using MethyLight, as well as in stool samples, using a much more sensitive technique as droplet digital PCR. Furthermore, we analysed expression levels of selected genes whose promoter CGIs were hypermethylated in CRC, detecting downregulation at mRNA and protein levels in CRC tissue for GRIA4, VIPR2, SPOCK1 and SLC6A3. Most of these genes were already lowly expressed in colon normal tissues supporting the idea that cancer DNA methylation targets genes already barely expressed in the matched normal tissues. Our study suggests *GRIA4* and *VIPR2* as biomarkers for early CRC diagnosis using stool samples and confirms downregulation of genes hypermethylated in CRC.

## 1. Introduction

Colorectal cancer (CRC) is the third most common cancer worldwide and one of the most frequent cause of cancer death. Early diagnosis has a relevant importance for its prevention and treatment [1]. Therefore, to improve diagnosis in the early stage of colorectal cancer, researchers focused on the identification of biomarkers at genetic and epigenetic levels [2,3,4,5,6]. CRC diagnosis requires examination of tissue collected during colonoscopy. However, colonoscopy is a costly and invasive procedure, thus screening tests on stool samples have been developed. These tests include faecal occult blood test (FOBT) and faecal immunochemical test (FIT) but, although they can offer indications for possible CRC, their specificity and sensitivity are still limited [7].

Methylation alterations occur early in tumour progression [4,6,8] predating genetic mutations, and can be detected in non-invasive matrices, such as stool and plasma samples [9]. Therefore, they represent extremely useful biomarkers for early CRC diagnosis. In fact, several commercial kits analysing methylation-based biomarkers are available. However, these kits have shown low specificity and sensitivity for the detection of early stages CRC and advanced adenomas, generating false positive and negative results [10,11,12]. Recently, our research group identified 74 altered CpG Islands (CGI) in both CRCs and adenomas confirming that methylation alterations are early events during CRC tumorigenesis [4]. As highlighted in our previous work, CGIs that undergo early methylation alteration are associated with genes involved in cell cross-talking and cell signalling pathways, such as membrane receptors, secreted signalling proteins and cell adhesion molecules. These data would suggest that the first modifications of the cell towards a carcinogenesis path involve the interaction of the cell with the other cells and with the surrounding microenvironment.

In this work, we analysed selected genes that may be not only promising biomarkers for early CRC detection but are also representative of the aforementioned most altered biological processes in colon tumorigenesis. In particular, we studied methylation status of *GRIA4* and *VIPR2*, gene and protein expression levels of *GRIA4*, *VIPR2*, *SPOCK1* and *SLC6A3* in 10 CRC tissue samples and their matched normal tissues. Moreover, we explored the possible usefulness of *GRIA4* and *VIPR2* associated CGIs as early CRC biomarkers in stool samples.

Among the selected biomarkers, the Glutamate Ionotropic Receptor AMPA Type Subunit 4 (*GRIA4*) encodes a homonym subunit of the AMPA tetrameric receptor complex [13]. The principal function of this receptor type, as a cationic ion channel, is mainly performed in the central nervous system, e.g., synaptic communication [14]. Although knockdown of *GRIA4* has been associated with dysregulation of genes involved in invasion and metastasis [15], its functional role in cancer is not fully elucidated. Interestingly, *GRIA4*-associated CGI has been found hypermethylated in CRC and adenomas [3,4,16].

The vasoactive intestinal peptide receptor 2 (*VIPR2*; alias *VPAC2*) encodes a homonym transmembrane protein that is associated with a guanine nucleotide binding protein. VIPR2 is activated through the interaction with the ligands VIP, PACAP-38 and -27 [17]. Specifically, VIPR2 receptor is involved in smooth muscle relaxation, exocrine and endocrine secretion and it has been associated with physiological processes such as the circadian activity and the immune response [17]. This type 2 receptor is expressed in the central nervous system, smooth muscle and blood vessels [17,18]. *VPAC2* has been poorly studied in cancer, but it has been detected in thyroid, gastric, lung and neuroendocrine tumours, among others [19,20,21].

The SPARC/Osteonectin, Cwcv and Kazal like domains proteoglycan 1 (*SPOCK1*) gene encodes a glycoprotein of the extracellular matrix whose principal role is in cell–cell and cell–matrix interactions. It has been reported that this gene is frequently altered in various tumour types such as lung, prostate and colon carcinomas [22,23,24]. Previous research demonstrated that SPOCK1 achieves cell cycle regulation through the PI3K/AKT pathway, but nevertheless its function is not well elucidated [24].

The Solute Carrier Family 6 Member 3 (*SLC6A3*) encodes a dopamine transporter that resorbs synaptic dopamine. Its function has been mainly investigated in psychiatric disorders [25] and Parkinson’s disease [26]. Concerning its role in human malignancies, *SLC6A3* is also a target gene of Hypoxia Inducible Factor 1 (HIF-1), and thus its expression has recently been studied in glioblastomas in response to oxygen decline [27]. However, some evidence describes the participation of dopamine receptors in other types of tumours [28]. In fact, there is no proof of the role of *SLC6A3* in colorectal cancer. Nevertheless, it has been reported that dopamine improves the efficacy of therapy in breast and colon cancer [29]. In a different context, hypermethylation was detected in dopamine pathway genes, including *SLC6A3*, as a consequence of a high fat and sugar diet suggesting a link between dopaminergic signalling and metabolic control [30].

Our work confirmed the methylation alterations of *GRIA4-* and *VIPR2*-associated CGIs in CRC tissues as well as non-invasive matrices such as stool samples. Moreover, our results showed a downregulation at mRNA and protein levels in tumour colon tissue for all the analysed biomarkers.

## 2. Results

### 2.1. Biomarkers’ Selection

Twenty-four CGIs fulfilled our selection criteria (see Materials and Methods), having an area under curve (AUC) higher than 0.95 in our discovery set [4] and colon adenocarcinoma validation set from The Cancer Genome Atlas (TCGA-COAD) (Table 1).

The four biomarkers’ selection was based on a combination of the above parameters but also on the fact that they were firstly discovered by our research group [4], and for their functional roles. Two CGIs (chr11:105481126-105481422 and chr7:158936507-158938492), respectively mapping on *GRIA4* and *VIPR2* gene promoters, were selected for the methylation analyses. *VIPR2* was selected for its functional role and the involvement of vasoactive intestinal peptide receptors in cancer [21], while *GRIA4* was chosen because it has previously shown methylation alterations in CRC tissues as well in plasma and stool samples [3,4].

**Figure 1:**
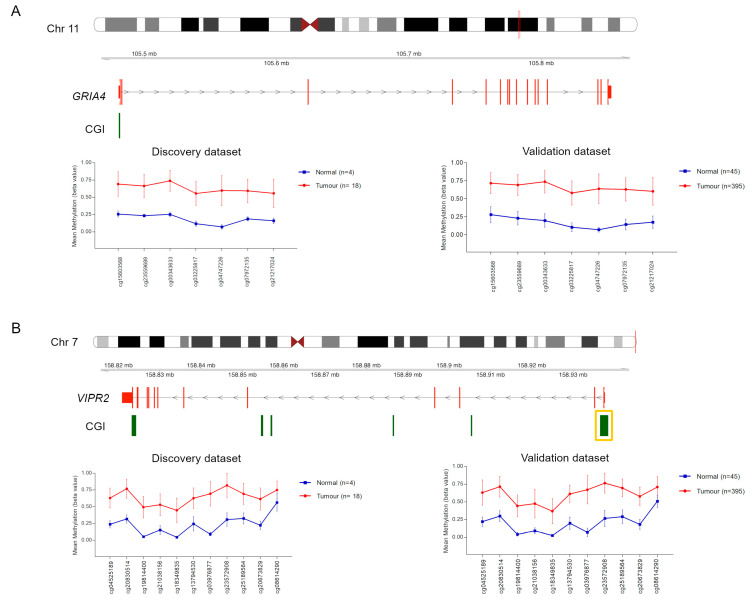
Methylation values obtained from our colorectal (CRC) discovery set and The Cancer Genome Atlas (TCGA) validation set. Genomic organization of *GRIA4* (**A**) and *VIPR2* (**B**), including the localization of exons and CGIs. Mean β values, resulting from the average of the samples (normal and tumour) of each probe mapping on the altered CGIs associated with *GRIA4* (**A**) and *VIPR2* (**B**). When more than one CGI is shown, the altered one is enclosed in a yellow box.

These two CGIs were hypermethylated in CRC samples in our discovery set and TCGA validation dataset (Figure 1).

### 2.2. Methylation Analyses

Further methylation analyses of *GRIA4* and *VIPR2* were conducted on 10 CRC paired tissue samples using MethyLight qPCR.

Methylation levels of tumour samples were compared to those of their respective normal samples.

*GRIA4* showed hypermethylation in 6/10 tumour samples, while *VIPR2* was hypermethylated in 7/10 tumour samples. For some tumour samples methylation levels of *GRIA4* (2/10 samples) or *VIPR2* (2/10 samples) were similar to their respective normal samples, while in other cases, methylation of *GRIA4* (2/10 samples) and *VIPR2* (1/10 sample) was not detectable, probably due to the low content of tumour cells in these tissue samples. (Table 2).

Tissue specimens were examined by a histopathologist to investigate the possible causes of these methylation differences among the analysed samples. Tumour samples with undetectable methylation or methylation levels similar to the respective normal samples showed low content of tumour cells. On the other hand, tumour samples with high methylation levels showed a high content of tumour cells.

To investigate whether these alterations can also be detected through a non-invasive procedure as biomarkers, their methylation levels were evaluated in stool samples from the same cohort by MethyLight.

*GRIA4* methylation was detected in 4/10 samples, while *VIPR2* methylation was detected in 7/10 samples (Table 2).

Since MethyLight might not be sensitive enough for the detection of traces of methylated DNA, the same samples were analysed using a much more sensitive technique—droplet digital PCR (ddPCR). This method allowed to detect methylation of both *GRIA4* and *VIPR2* in 9/10 samples (Table 2).

### 2.3. mRNA Expression Study

**Figure 2:**
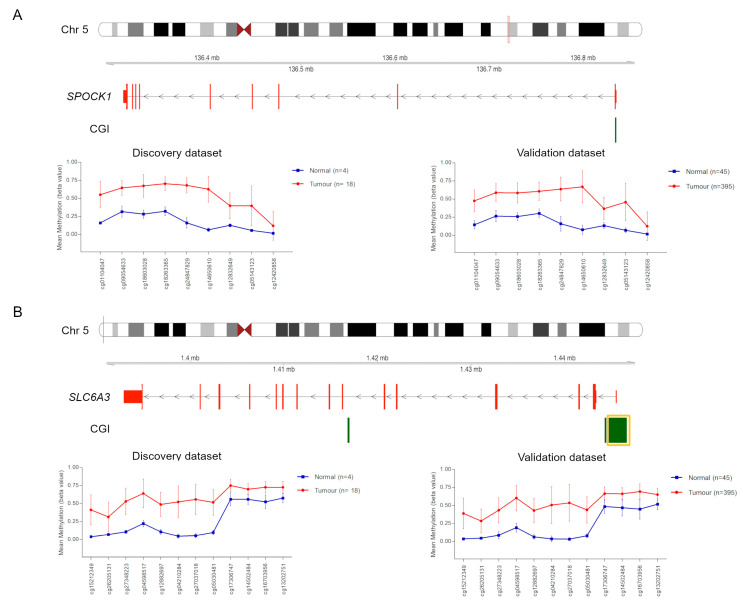
Methylation values obtained from our CRC discovery set and TCGA validation set. Genomic organization of *SPOCK1* (**A**) and *SLC6A3* (**B**), including the localization of exons and CGIs. Mean β values, resulting from the average of the samples (normal and tumour) of each probe mapping on the altered CGIs associated with *SPOCK1* (**A**) and *SLC6A3* (**B**). When more than one CGI is shown, the altered one is enclosed in a yellow box.

The same 10 CRC paired tissue samples were analysed for gene expression of *GRIA4*, *VIPR2* and two additional genes, *SPOCK1* and *SLC6A3*, whose CGIs were hypermethylated in our CRC discovery set and successfully validated in TCGA dataset (Figure 2 and Table 1) [4]. Unfortunately, we did not have enough material to conduct further methylation analyses for *SPOCK1* and *SLC6A3*.

**Figure 3:**
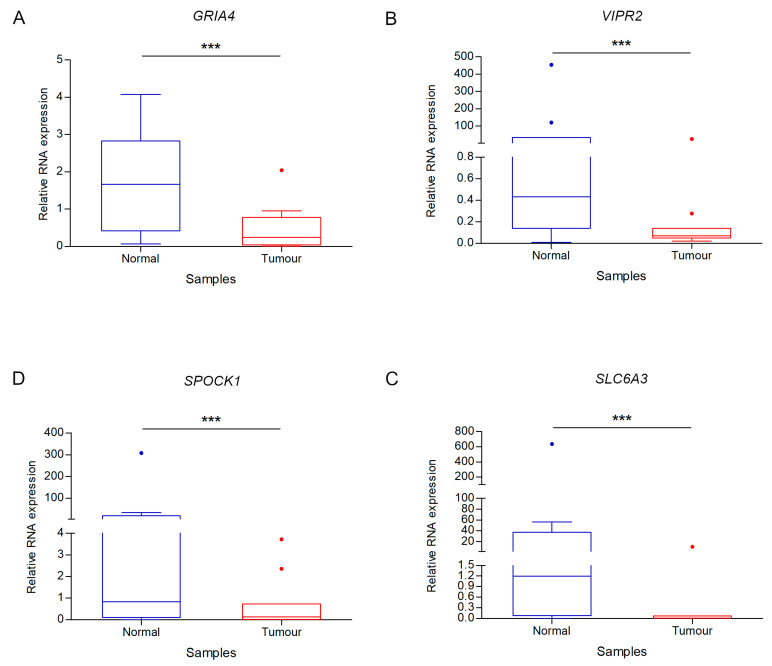
Differential gene expression analysis between tumour and normal samples. Box plot fold change values of *GRIA4* (**A**), *VIPR2* (**B**), *SPOCK1* (**C**) and *SLC6A3* (**D**) for CRC and control samples. *** indicates *p* value < 0.0001.

The mean expression of each of the four genes was markedly lower in tumour than in normal tissue (Figure 3). Of note, in our experimental study *SLC6A3* revealed the greatest average expression difference between tumour and normal tissues (Figure 3).

**Figure 4:**
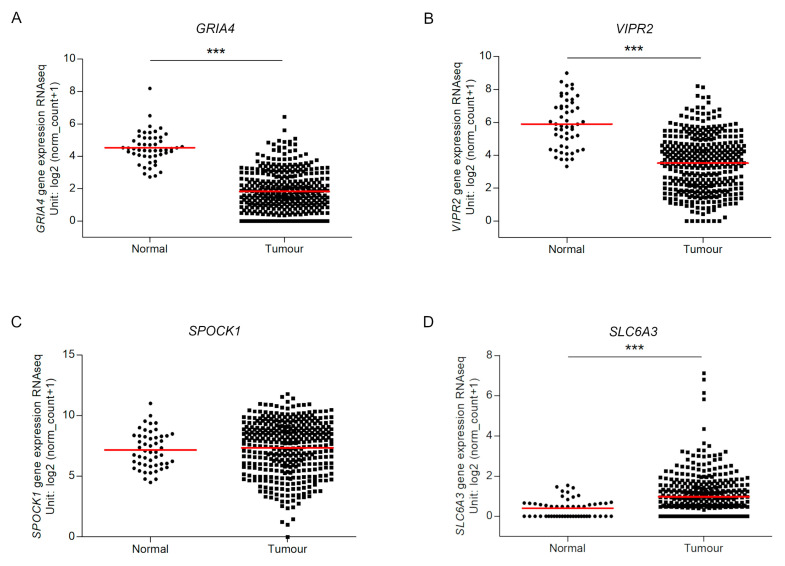
RNA expression of *GRIA4* (**A**), *VIPR2* (**B**), *SPOCK1* (**C**) and *SLC6A3* (**D**) in TCGA dataset. Tumour and normal expression of the four biomarkers. *** indicates *p* value < 0.0001.

We investigated the gene expression of the four genes between tumour and normal tissues using TCGA expression data obtained by RNA-seq. This analysis confirmed the significant lower expression level of *GRIA4* and *VIPR2* in tumour tissues than in normal ones. In disagreement with our experimental data, *SLC6A3* expression analysis revealed higher levels in tumours and *SPOCK1* presented similar expression levels in both tissues (Figure 4).

### 2.4. Protein Expression Study

**Figure 5:**
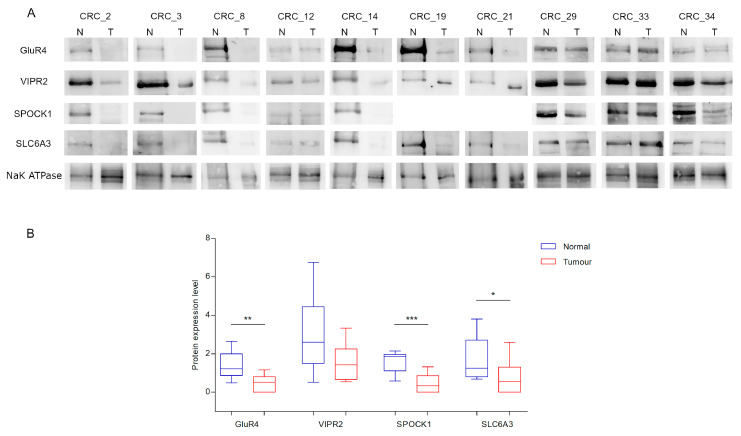
Protein expression level for each paired sample. (**A**) Representative blots of GluR4, VIPR2, SPOCK1 and SLC6A3 protein expression in 10 CRC paired tissues samples. NaK ATPase was used as loading control. (**B**) Box plots of GluR4, VIPR2, SPOCK1 and SLC6A3 expression. Asterisks indicate statistically significant differences (* *p* value < 0.05, ** *p* value < 0.01, *** *p* value < 0.001). For the SPOCK1 expression study, there was not enough protein lysate for samples 19 and 21.

Protein expression level of the four biomarkers were analysed by Western Blot. SPOCK1, SLC6A3 and GluR4 were significantly low-expressed in tumour respect to normal tissues, following the same pattern of the mRNA expression level (Figure 5 and Figure S1). On the contrary, VIPR2 did not show statistically significant differences of expression between tumour and normal tissues.

## 3. Discussion

Colorectal cancer develops through the accumulation of genetic and epigenetic aberrations. Methylation alterations in CRC could be used as biomarkers for early diagnosis, and/or prognostic and predictive markers to improve therapy. In fact, our research group identified a panel of 74 altered CGIs that discriminates tumour and adenoma tissues from normal tissues [4]. In the current work, we focused on four genes, *GRIA4*, *VIPR2*, *SPOCK1* and *SLC6A3*, whose CGIs were hypermethylated in CRC in our previous study. Further methylation analyses were performed in 10 paired samples for *GRIA4* and *VIPR2*, confirming their alteration in CRC. The absence of hypermethylation of *GRIA4* and *VIPR2* associated CGIs in CRC tissue samples with low content of tumour cells highlights the high specificity of these two biomarkers for CRC detection. To note, sample collection during surgery is a rigorous step that must be carefully controlled and may lead to possible false results. In fact, in the current study a methodological error in tissue sample collection caused apparent contradictory results. However, this contradiction has been uncovered by an accurate histopathological analysis. We analysed stool samples from the same cohort of CRC patients. Stool samples from healthy individuals were not included in this study. Nevertheless, it is expected that they have an even lower number of methylated targets in a high background of unmethylated molecules compared to stool samples of CRC patients. To note, in CRC patients the concentration of exfoliated colonocytes, released in faeces, is 4.5-fold higher than in healthy individuals [31]. Importantly, our results showed that MethyLight gave false negatives in stool samples. Nevertheless, we were able to confirm that using a very sensitive method, such as ddPCR, these two methylation alterations were detectable in all except one stool sample. The lack of methylation positivity in this sample could be due to the loss of tumour DNA from the faeces suggesting the high specificity of our biomarkers for tumour DNA. In fact, stool samples are collected from the resected tumour tissues after their washout to clean the specimens for the pathology examination. Moreover, it must be considered that the patients follow a special diet including laxative drinks to clean the bowel before surgery [32].

Therefore, the detection of these methylation-based biomarkers using ddPCR would allow an early diagnosis of CRC and the follow-up of patients after tumour surgical resection and/or treated with chemotherapy and/or radiotherapy. In fact, ddPCR can overcome the technical challenges (i.e., poor DNA quality, presence of contaminating DNA and PCR inhibitors) responsible for a reduced performance of MethyLight for the detection of low copies of methylated DNA in such samples, being 25-fold more sensitive than conventional MethyLight [33]. Our results confirm that ddPCR is the gold standard method for detecting low copies of tumour DNA in non-invasive matrices.

Given that FOBT and other biomarkers screening tests recently introduced in the sanitary system give a high number of false positive results [7,9], our methylation biomarkers can potentially improve the current diagnosis system, reinforcing our previous results for *GRIA4*-associated CGI [3,4].

Moreover, we studied the transcript and protein expression levels of the four selected biomarkers, whose associated CGIs are located in promoter regions, demonstrating their downregulation in tumour tissues. Not all samples reflect a direct correlation between RNA and protein expression given that factors, such as post-transcriptional modification, translational regulation and protein half-lives, regulate gene expression. However, the mean values showed a correlation between transcript and protein expression levels. These results confirmed the association between promoter hypermethylation and gene downregulation already reported, considering methylation as a mechanism for gene transcriptional inactivation in cancer [34,35]. Moreover, the expression levels of most of the genes studied in this work, were already low in colon normal tissues (GTEx data, https://www.gtexportal.org) supporting the idea that methylation in cancer targets genes barely expressed in tissues where tumour arises [4,36,37,38]. Interestingly, our study shows that these genes are further repressed in tumour tissues that can be observed only by means of a targeted gene expression analysis as previously reported [4,36,39]. This further downregulation can be detected only by using methods such as qRT-PCR. In fact, the background levels of hybridization to probes in gene expression microarray or the low sequencing depth for low expressed genes in RNA-seq do not allow us to detect small gene expression changes of low-expressed genes [4,40,41].

These might be the possible reasons for the lack of validation of our gene expression results for *SPOCK1* and *SLC6A3* in TCGA RNA-seq data, unlike *GRIA4* and *VIPR2*.

The current study confirmed *GRIA4* methylation alteration in CRC [3,4] and gives further support of its possible role in tumour by means of the altered expression detected at the RNA and protein level.

There is little evidence of the role of the VPAC receptors in cancer although their expression was already reported in a variety of tumours. In particular, VPAC1 receptors are expressed in malignant epithelial neoplasms, while expression of VPAC2 receptors has mainly been found in some leiomyomas and gastrointestinal stromal tumours [18,42,43,44]. Our results demonstrated protein and transcript expression of *VIPR2* in normal and tumour tissue enriching the knowledge of its possible involvement in CRC. However, we did not detect a statistically significant reduction of VIPR2 at protein level in tumour tissues, probably for the large variability among the samples, although a tendency towards downregulation can be observed.

Our finding that *SPOCK1* is downregulated in CRC is in contrast with TCGA data and with previous research that demonstrated its overexpression in colon cancer [24,45] and in other tumour types [21,23]. In TCGA data, this gene was similarly expressed in tumour and normal samples. Moreover, Sanz-Pamplona et al. (2014) showed that SPOCK1 was upregulated in the normal mucosa adjacent to CRC tissue (minimum distance of 10 cm) compared to colon mucosa from healthy donors. However, its expression in the normal mucosa samples from CRC patients was similar to that of the tumour. In fact, at the molecular level peritumoural cells resembled more malignant cells than normal cells, i.e., the surrounding environment could already present alterations similar to the tumour tissue. Protein–protein network analysis showed that SPOCK1 is a protein secreted by adjacent mucosa and interacts with a receptor in tumour [45]. Of note, some of our tumour samples have shown higher SPOCK1 expression than their matched-normal sample, indicating that its expression is highly variable. Therefore, further studies to clarify these discordant results are needed.

The expression of the dopamine transporter *SLC6A3* was significantly downregulated in our CRC samples, both at mRNA and protein levels. In contrast, this gene resulted upregulated in CRC in TCGA data. However, gene expression values of tumours markedly overlap those of normal samples in TCGA dataset. The discrepancy with our results might be related to the low expression of this gene in the colon (GTEx data: about 0.8 TPM) and even reduced expression in tumours that might be not detected by techniques such as RNA-seq and this result should be validated in a larger sample cohort by ultrasensitive methods such as ddPCR. SLC6A3 possible role in cancer might be due to the well-known protective effects of dopamine in tumours [28]. Previously, it was reported that Slc6a3 was hypermethylated in the promoter region in response to high-fat-sucrose diet and downregulated in the nervous system of prenatally stressed female rats and murine models of diet-induced obesity [46,47]. In addition, methylation aberrations at *SLC6A3* gene were related to triglyceride level and obesity in humans [30,48]. Hence, the hypermethylation event at the promoter region of *SLC6A3*, detected previously by our group [4], and its low expression in CRC, reported in the current study, could suggest an alteration in metabolism, such as lipogenic pathways, and probably a oxidative stress environment leading to malignancy transformation [49]. This statement can also be supported by the involvement of *SLC6A3* in response to hypoxia, in glioblastomas, as one of the target genes of HIF-1 [27].

In summary, our work was focused on the methylation and expression analyses of *GRIA4*, *VIPR2*, *SPOCK1* and *SLC6A3*, belonging to gene families involved in the crosstalk between tumour cells and the environment. From our point of view, the identified hypermethylation and reduced expression of these normally low expressed genes but further downregulated in cancer, underline their functional involvement in a defined program of gene silencing during tumour transformation. This is in agreement with the “epigenetic switching” concept allowing the maintenance of a stable and permanent repression of genes important for cell proliferation and tumorigenesis leading to the restoring of a stem-cell-like state [50,51]. Of note, epigenome editing approaches may allow to re-establish the normal methylation and gene expression patterns of these genes, possibly reverting the tumour phenotype to that characteristic of a normal cell.

## 4. Materials and Methods

### 4.1. Tissue Samples

Tumour and matched-normal fresh-frozen (FF) tissue samples of 10 CRC patients were collected from the Department of General Surgery of the University of Cagliari (Italy). Normal samples were taken at a distance >10 cm from the neoplastic tissue. All the biological samples analysed were obtained with written informed consent signed from patients and ethical approval granted by the relative Ethics Committee.

Histopathological analysis was performed for all the tissue samples. Frozen section slides underwent standard haematoxylin and eosin (H&E) staining. Microscope images were acquired of each individual slide.

Patients’ clinical data are reported in Table 3.

### 4.2. Stool Samples

Stool samples were collected from the same cohort of ten CRC patients taken intraoperatively from the bowel resection specimen. All stools samples were immediately frozen after collection and stored at −80 °C until being processed.

### 4.3. Marker Selection

Biomarkers were selected from a panel of 74 altered CGIs, previously identified from a genome-wide methylation study of 18 primary CRCs and four matched peritumoural samples, 21 colorectal adenomas and three matched-normal intestinal mucosa samples by our research group [4].

**Figure 6:**
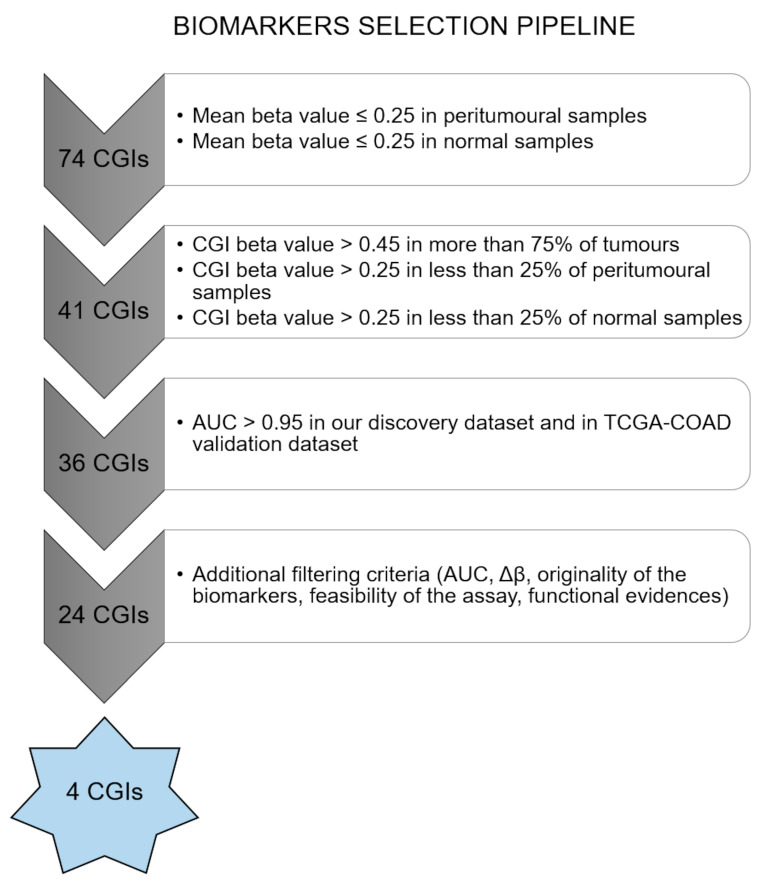
Biomarkers’ selection pipeline. Pipeline for biomarkers selection using methylation data from our previous CRC discovery set and from TCGA-COAD dataset.

Methylation data from this cohort (discovery set) and methylation data of TCGA-COAD were analysed for the biomarkers selection using the following pipeline summarized in Figure 6. In the first step, CGIs for which mean beta value was lower than 0.25 in peritumoural and normal samples were retained. Subsequently, only CGIs with a beta value higher than 0.45 in at least 75% of tumours and a beta value higher than 0.25 in not more than 25% of peritumoural and normal samples were selected. Receiver operating characteristic (ROC) curves for the 36 selected biomarkers were performed by R “ROCR” package. A group of 24 CGIs showed an AUC higher than 0.95 in our cohort and in TCGA-COAD.

Four biomarkers were finally selected for a combination of parameters (AUC, Δβ), functional roles and because they were originally identified by our group [4]. Additional filtering criteria included feasibility of the assay. Further methylation analyses were conducted only for two out of the four selected biomarkers (*GRIA4* and *VIPR2*), because of the lack of biological material for the other assays.

### 4.4. Methylation Analyses

Genomic DNA was extracted from tumour and peritumoural tissue using the DNeasy Blood & Tissue Kit (Qiagen, Hilden, Germany). Stool DNA extraction was performed using the QIAamp DNA Stool Mini Kit (Qiagen, Hilden, Germany) according to the manufacturer’s instructions.

DNA samples were bisulfite converted using EZ DNA Methylation Gold Kit TM (Zymo Research, Irvine, CA, USA) according to the manufacturer’s instructions. Quality control and quantification of DNA were performed before and after bisulfite conversion.

*GRIA4* and *VIPR2* methylation was assessed by using MethyLight qPCR [52] in tissue and stool samples. The reference repetitive element Alu was used in a methylation-independent control reaction to normalize the amount of DNA input. Primers and probes were designed using Beacon Designer™ (Premier Biosoft, San Francisco, CA, USA) (Table 4). The probes were labelled with the 6-Carboxyfluorescein (6-FAM) fluorophore at the 5′ end. A primer-probe mix containing 300 nM of each primer and 100 nM of the probe was prepared. Each assay was performed in triplicate using: 15 μL of TaqMan Genotyping Master mix (Applied Biosystems, Foster City, CA, USA), 4.5 μL of primer-probe mix, 5 μL of bisulfite-converted DNA (10 ng/μL) and 5.5 μL of RNase-free water. A fully-methylated DNA (Human Methylated & Non-Methylated (WGA) DNA Set; Zymo Research, Irvine, CA, USA) was used as a positive control for the reaction. The experiment was conducted on a DNA Engine Opticon 2 Real-Time Cycler (Bio-Rad, Hercules, CA, USA) using the following thermal conditions: initial PCR activation step at 95 °C for 10 min, followed by 50 cycles of denaturation step at 95 °C for 15 s and annealing/extension step at 60 °C for 60 s.

A comparison between CRC and their matched-normal samples have been performed. CRC samples were considered as hypermethylated if delta Ct (mean Ct normal − mean Ct tumour) > 2 and as hypomethylated if delta Ct (mean Ct normal − mean Ct tumour) < −2.

Methylation of the two biomarkers was also evaluated by ddPCR in the stool samples from CRC patients using the same primers and probes used in the MethyLight assay (Table 4).

ddPCR reactions containing 2 × ddPCR Supermix for probes (Bio-Rad, Hercules, CA, USA), forward and reverse primer (900 nM), probe (250 nM) and 2 to 5 μL bisulfite-converted DNA in a final volume of 20 μL were partitioned into ~20,000 oil-emulsified droplets per well and replicated in three wells using a Bio-Rad QX200 droplet generator (Bio-Rad, Hercules, CA, USA). The droplets were transferred into 96-well plates and PCR was performed using the following conditions: 10 min at 95 °C, 40 cycles of 30 s at 95 °C followed by 60 s at 60 °C, then 5 min at 4 °C, 5 min at 95 °C. Plates were read on a Bio-Rad QX200 droplet reader (Bio-Rad, Hercules, CA, USA).

Data were analysed using the QuantaSoft 1.7.4 software (Bio-Rad). The droplet counts (positive or negative) from all replicated wells were combined to yield a ‘merged’ well. Concentration values (number of copies/μL) and Poisson confidence intervals were computed for each “merged” well.

### 4.5. mRNA Expression Analysis

Total RNA was extracted from tumoural and normal samples using the RNeasy Mini Kit (Qiagen, Hilden, Germany) following manufacturer’s protocol. An aliquot of 1μg RNA/sample was retrotranscribed using the High Capacity Kit (Applied Biosystems, Carlsbad, CA, USA). Gene expression was evaluated by qPCR using SsoAdvanced™ Universal SYBR^®^ Green Supermix (Bio-Rad, Hercules, CA, USA) for each gene tested, *GRIA4*, *SLC6A3*, *SPOCK1* and *VIPR2*, and for the endogenous gene *TFRC*. Primers sequences can be found in Table 5. PCR conditions were primary denaturation at 95 °C for 2 min followed by 50 cycles of denaturation at 95 °C for 15 s and annealing/extension at 60 °C for 1 min. After the amplification cycles, melting curves were produced by increasing the temperature from 65 °C to 95 °C holding each temperature for 5 sec and reading fluorescence every 0.5 °C.

The transcript levels were quantified using the ΔΔCT method. Statistical analyses were done by comparing the average ΔCt of the sample groups of interest using Welch’s *t*-test. We considered upregulation or downregulation when there existed statistical significant differences in the expression level of tumour tissues compared to normal tissues, whereas the expression between tissues was considered similar when the statistical analysis was not significant.

### 4.6. Protein Expression Analysis

The protein expression level of SLC6A3, SPOCK1, VIPR2 and GluR4 was studied by Western blot. Proteins from colon tissue were isolated using the Membrane Protein Extraction Kit (Mem-PERTM Plus, Thermo Fisher Scientific, Waltham, MA, USA) following the manufacturer’s instructions. Protein extracts were fractioned by 10% SDS-polyacrylamide gel and separated at 100 V during 90 min. Then, the proteins were transferred onto 0.45 µm nitrocellulose membrane, blocked in 5% milk overnight at 4 °C and then incubated 2 h with primary antibody GluR4 (0.5 µg/mL, PA5-18931, Thermo Fisher Scientific, Waltham, MA, USA), SPOCK1 (1 µg/mL, MA5-24039, Thermo Fisher Scientific, Waltham, MA, USA), VIPR2 (0.5 µg/mL, AB2266, Millipore, Burlington, MA, USA) and SLC6A3 (1 µg/mL, SAB2502027, Sigma-Aldrich, St. Louis, MO, USA). After hybridization with primary antibody, the membrane was washed with Tris-buffered saline containing Tween-20 three times, then incubated with horseradish peroxidase-labelled secondary antibody (Jackson ImmunoResearch, Ely, UK) for 1 h at room temperature and washed with Tris-buffered saline containing Tween-20 three times. Final detection was performed with ECL Chemiluminescent Western blotting reagents (Bio-Rad, Hercules, CA, USA). An antibody against NaK ATPase (1:40000, Ab76020, Abcam, Cambridge, UK) was used for gel-loading control. Unfortunately, there was not enough protein lysate of the subjects 19 and 21 to study the expression level of SPOCK1.

Western blot signals were quantified using ImageJ program. The intensity of each band was normalized with respect to that of NaK ATPase. The assay was performed in duplicate. Statistical differences between tumour and normal samples were calculated using Welch’s *t*-test.

### 4.7. Validation Analyses

Methylation and RNA-seq data from TCGA, including colorectal samples, were used to validate our methylation and gene expression results. Statistical differences between tumour and normal samples were calculated using Welch’s *t*-test.

## 5. Conclusions

In conclusion, our study suggests *GRIA4* and *VIPR2* as biomarkers for CRC early detection by non-invasive methods, in particular in stool samples. Further studies are needed to explore methylation detection of these two biomarkers in stool samples from population subjected to CRC screening and prove their specificity and sensitivity for early diagnosis. Moreover, we confirm the downregulation of genes hypermethylated in tumours and suggest the role of these genes in CRC although functional studies are needed to support this hypothesis.

## Figures and Tables

**Table 1:** Selected CpG islands showing an area under curve (AUC) > 0.95 in our discovery set and in TCGA-COAD validation set.

CGI	Gene	Δβ Discovery Set	AUC Discovery Set	Δβ Validation Set	AUC Validation Set
chr2:182321761-182323029	*ITGA4*	0.37	1.00	0.35	0.96
chr4:156129168-156130209	*NPY2R*	0.27	1.00	0.3	0.97
chr4:157997166-157997686	*GLRB*	0.30	1.00	0.36	0.97
chr4:107956555-107957453	*DKK2*	0.32	0.97	0.33	0.97
chr5:136834016-136835146	*SPOCK1*	0.29	1.00	0.33	0.98
chr5:140864527-140864748	*PCDHGA4*	0.35	1.00	0.38	0.97
chr5:1444678-1446648	*SLC6A3*	0.26	1.00	0.29	0.98
chr5:178016558-178017670	*COL23A1*	0.29	0.98	0.32	0.96
chr5:159399004-159399928	*ADRA1B*	0.25	0.97	0.31	0.97
chr6:159589636-159591319	*FNDC1*	0.33	1.00	0.33	0.97
chr6:73330942-73333109	*KCNQ5*	0.36	1.00	0.33	0.96
chr7:28448716-28450028	*CREB5*	0.27	1.00	0.27	0.96
chr7:158936507-158938492	*VIPR2*	0.33	0.96	0.35	0.96
chr8:97505747-97507607	*SDC2*	0.29	1.00	0.36	0.96
chr8:75896528-75897116	*CRISPLD1*	0.21	0.96	0.27	0.97
chr10:15761423-15762101	*ITGA8*	0.30	0.96	0.35	0.97
chr11:105481126-105481422	*GRIA4*	0.40	1.00	0.41	0.96
chr11:133938850-133939681	*JAM3*	0.30	1.00	0.29	0.97
chr12:117798076-117799448	*NOS1*	0.25	0.97	0.27	0.96
chr13:110958891-110960590	*COL4A1*	0.33	0.96	0.37	0.98
chr16:23846941-23848102	*PRKCB*	0.25	0.98	0.34	0.97
chr19:48918115-48918340	*GRIN2D*	0.33	1.00	0.38	0.96
chr21:28337856-28340237	*ADAMTS5*	0.29	1.00	0.31	0.99
chr22:33453892-33454505	*SYN3*	0.28	0.97	0.32	0.97

AUC: Area under curve; CGI: CpG island.

**Table 2:** *GRIA4* and *VIPR2* methylation analyses results.

	*GRIA4*			*VIPR2*		
	Tumour Tissue Sample	Stool Sample MethyLight	Stool Sample ddPCR	Tumour Tissue Sample	Stool Sample MethyLight	Stool Sample ddPCR
**CRC_2**	Hyper methylated	Methylated	Methylated	Hyper methylated	Methylated	Methylated
**CRC_3**	Hyper methylated	Methylated	Methylated	Hyper methylated	Methylated	Methylated
**CRC_8**	Undetectable methylation	Undetectable methylation	Methylated	Undetectable methylation	Methylated	Methylated
**CRC_12**	Undetectable methylation	Undetectable methylation	Methylated	Hyper methylated	Undetectable methylation	Methylated
**CRC_14**	Not differentially methylated	Undetectable methylation	Methylated	Hyper methylated	Methylated	Methylated
**CRC_19**	Hyper methylated	Methylated	Methylated	Not differentially methylated	Methylated	Methylated
**CRC_21**	Hyper methylated	Undetectable methylation	Undetectable methylation	Hyper methylated	Undetectable methylation	Undetectable methylation
**CRC_29**	Not differentially methylated	Methylated	Methylated	Hyper methylated	Methylated	Methylated
**CRC_33**	Hyper methylated	Undetectable methylation	Methylated	Hyper methylated	Methylated	Methylated
**CRC_34**	Hyper methylated	Undetectable methylation	Methylated	Not differentially methylated	Undetectable methylation	Methylated

CRC: Colorectal cancer.

**Table 3:** Clinical characteristics of CRC patients.

Sample ID	Tumour Location	Stage at Diagnosis	Mucinous Histology	Lymphovascular Invasion	Grade	Ulcerative Neoplasia
**CRC_2**	Left colon	I	NO	NO	G2	NO
**CRC_3**	Right colon	III	NO	YES	G2	YES
**CRC_8**	Rectum	IV	YES	YES	G2	NO
**CRC_12**	Right colon	0	NO	YES	G1	NO
**CRC_14**	Rectum	III	YES	YES	G3	NO
**CRC_19**	Right colon	II	YES	YES	G2	YES
**CRC_21**	Transversal colon	II	NO	YES	G2	NO
**CRC_29**	Right colon	II	NO	YES	G2	NA
**CRC_33**	Right colon	IV	NO	YES	G2	NA
**CRC_34**	Rectum	III	NO	YES	G2	YES

CRC: Colorectal cancer.

**Table 4:** Primers and probe sequences for MethyLight and ddPCR assay.

Target	Forward Primer (5′–3′)	Reverse Primer (5′–3′)	Probe (5′–3′)
*GRIA4*	GGGTTGGTGTAGGTTTGTT	CTCCCCCCTTACTTTCTCACATACACACAA	AACGCCGCGACCGCCACAC
*VIPR2*	TCGGTTTCGAGTAGAGAGAATTGG	AAACAAATACAAACGACCGCAAAA	CCCTTCCGAACGCACACCTAACCC
Alu	GGTTAGGTATAGTGGTTTATATTTGTAATTTTAGAT	ATTAACTAAACTAATCTTAAACTCCTAACCTCA	CCTACCTTAACCTCCC

**Table 5:** Primers for quantitative real-time polymerase chain reaction (qRT-PCR) assay.

	Gene	Forward Primer (5′–3′)	Reverse Primer (5′–3′)
**CRC** **gene expression assay**	*GRIA4*	TCATGTGGACAACATTGAGACA	ATCATAGAGTCCAAAAATGGCAAA
	*VIPR2*	GTCTCTTGCAACAGGAAGCA	TCTCAGGATGAAGGACAGGAA
	*SLC6A3*	CCATACTGAAAGGTGTGGGCT	AGAAGAGATAGTGCAGCGCC
	*SPOCK1*	AGGTAAAATGCAGCCCTCACA	TTCCCCTTCTTTTGCCTGGG
	*TFRC*	GGCACAGCTCTCCTATTGAAAC	CAAAGTCTCCAGCACTCCAACT

CRC: Colorectal cancer.

## Supplementary Materials

The following are available online at https://www.mdpi.com/1422-0067/21/12/4494/s1.
