# Supplementary figures and images for: Colorectal Cancer Early Detection in Stool Samples Tracing CpG Islands Methylation Alterations Affecting Gene Expression
Source: Int J Mol Sci. 2020 Jun 24;21(12):4494. doi: 10.3390/ijms21124494 (PMC7349989; doi:10.3390/ijms21124494)

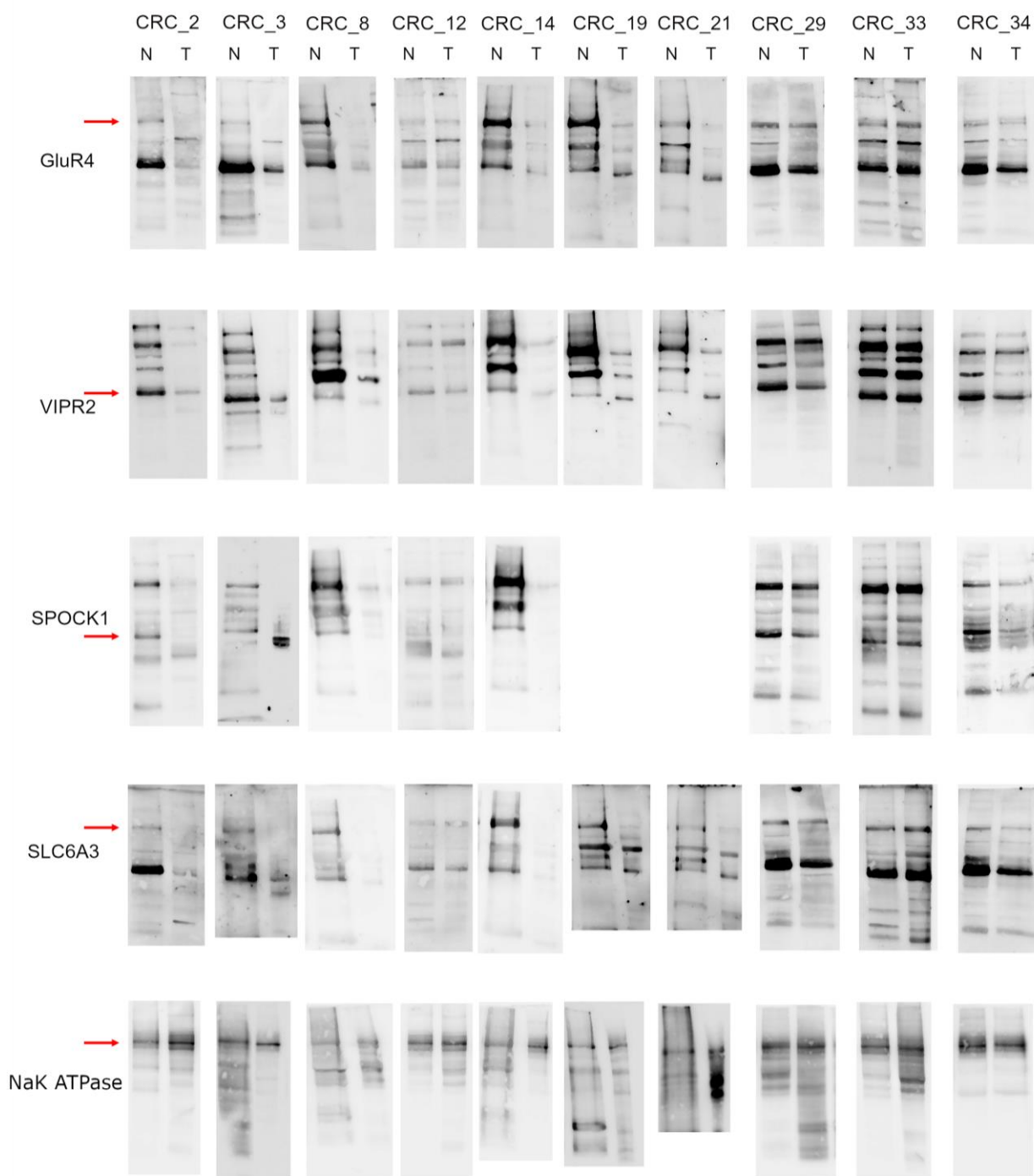

**Figure S1.** Full lanes of Western blot study. Red arrows indicate molecular weight.

Supplement: Supplementary file 1 [file ijms-21-04494-s001.pdf]
